# 3D-printed microrobots from design to translation

**DOI:** 10.1038/s41467-022-33409-3

**Published:** 2022-10-05

**Authors:** Sajjad Rahmani Dabbagh, Misagh Rezapour Sarabi, Mehmet Tugrul Birtek, Siamak Seyfi, Metin Sitti, Savas Tasoglu

**Affiliations:** 1 Department of Mechanical Engineering, Koç University, Sariyer, Istanbul, 34450 Turkey; 2 Koç University Arçelik Research Center for Creative Industries (KUAR), Koç University, Sariyer, Istanbul, 34450 Turkey; 3 Koc University Is Bank Artificial Intelligence Lab (KUIS AILab), Koç University, Sariyer, Istanbul, 34450 Turkey; 4 Physical Intelligence Department, Max Planck Institute for Intelligent Systems, Stuttgart, 70569 Germany; 5 Koç University Research Center for Translational Medicine, Koç University, Sariyer, 34450 Istanbul, Turkey; 6 Institute of Biomedical Engineering, Boğaziçi University, Çengelköy, Istanbul, 34684 Turkey

**Keywords:** Design, synthesis and processing, Translational research, Sensors and biosensors

## Abstract

Microrobots have attracted the attention of scientists owing to their unique features to accomplish tasks in hard-to-reach sites in the human body. Microrobots can be precisely actuated and maneuvered individually or in a swarm for cargo delivery, sampling, surgery, and imaging applications. In addition, microrobots have found applications in the environmental sector (e.g., water treatment). Besides, recent advancements of three-dimensional (3D) printers have enabled the high-resolution fabrication of microrobots with a faster design-production turnaround time for users with limited micromanufacturing skills. Here, the latest end applications of 3D printed microrobots are reviewed (ranging from environmental to biomedical applications) along with a brief discussion over the feasible actuation methods (e.g., on- and off-board), and practical 3D printing technologies for microrobot fabrication. In addition, as a future perspective, we discussed the potential advantages of integration of microrobots with smart materials, and conceivable benefits of implementation of artificial intelligence (AI), as well as physical intelligence (PI). Moreover, in order to facilitate bench-to-bedside translation of microrobots, current challenges impeding clinical translation of microrobots are elaborated, including entry obstacles (e.g., immune system attacks) and cumbersome standard test procedures to ensure biocompatibility.

## Abstract (web summary)

Microbots have attracted attention due to an ability to reach places and perform tasks which are not possible with conventional techniques in a wide range of applications. Here, the authors review the recent work in the field on the fabrication, application and actuation of 3D printed microbots offering a view of the direction of future microbot research.

## Introduction

The emerging science of machines and robots fabricated on the scale of micro- and nano-meters (micro- and nano-robots) has advanced immensely in the last decade^1,2^. While novel additive manufacturing methods (i.e., three-dimensional (3D) printing techniques) are surpassing size-related limitations, the functionalities of these robots have advanced owing to the use of smart materials (i.e., materials designed to respond to a certain condition such as specific pH or protein level), more accurate actuation techniques (i.e., on- and off-board methods), and integration with physical intelligence (PI) as well as artificial intelligence (AI). Accordingly, these robots are becoming one of the emerging contrivances for biomedical applications, attaining their position as the next potential paradigm changer in minimally invasive medicine^3^ (e.g., microsurgeries^4^ as well as detection, manipulation, assembly, and isolation of objects^5,6^), targeted cell/drug deliveries^7–10^, and maneuverable navigation in viscous mediums^11^ (e.g., biological fluids such as blood) for imaging/scanning purposes^12–17^.

By definition, 3D printers produce objects in a layer-by-layer manner based on a computer-aided design (CAD)^18–22^. 3D printing has significantly contributed to different biomedical fields ranging from microfluidics to lab/organ-on-chip technologies^23–30^. In comparison with the conventional microrobot fabrication methods (e.g., lithography methods^31,32^, deposition techniques using electrochemistry^33–35^ or physical vapors^36,37^, assembly techniques^38^, rolled-up technology^39^, electroless plating^40^, and strain engineering method^41^), 3D printing technologies offer a relatively cost-efficient process with rapid turn-around intervals between design modifications. Besides, a wide range of materials can be 3D printed, including metals^42,43^, polymers (e.g., plastics and hydrogels)^44–49^, bioinks (i.e., biocompatible materials with/without embedded cells)^50–52^, and composites^53–55^. Therefore, comparatively high accessibility and a higher level of reproducibility reinforce the position of 3D printing as the emerging method for microrobot production even for users without superb micromanufacturing skills^1,56,57^.

**Fig. 1:**
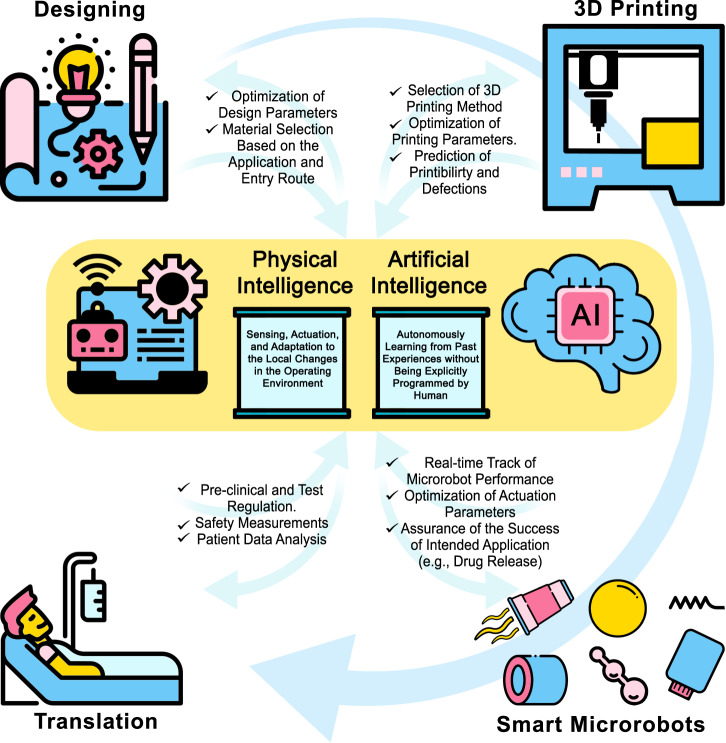
A schematic view of a microrobot from bench to bedside. The design and material-selection process can be assisted by AI and PI to design an application-specific device. AI can also improve the 3D printing process by optimizing printing parameters to reduce printing defects. In the application phase, AI can help clinicians to track microrobots and enhance maneuverability by tuning actuation parameters to ensure proper functionality. On the other hand, PI enables microrobots to sense different stimuli in their environment and respond to those stimuli independently (e.g., drug release at a particular pH level in the target site). In order to translate proof-of-concept microrobots to clinical medical devices, new microrobots must undergo standard tests to ensure safety for short- and long-term uses in the human body. However, these tests require cumbersome and costly procedures, which delays the rapid translation of microrobots. Thus, besides developing new materials, fabrication methods, and actuation modalities, more straightforward test procedures should be proposed to truncate the current test process while maintaining safety factors. Some elements in Fig. 1 have been designed using resources from Flaticon.com.

From a future perspective, AI not only can accelerate the design of a microrobot by optimizing design parameters more accurately than a human expert (e.g., determining optimum dimensions to minimize swimming friction in certain biofluids), but it also can play a role in the material-selection based on the chemical properties of the target site^1,58,59^. Besides, AI can be utilized to predict the printability of a design and tune 3D printing parameters to achieve the best printing possible (e.g., by adjusting light intensity (in light-induced methods) or pressure/temperature (in extrusion-based methods)). Following production, AI would facilitate the control of microrobots in vitro*/*vivo by adjusting the actuation parameters to ensure that microrobots will reach the target site despite unpredicted changes in the environment (e.g., unpredicted change of blood flow rate in a vessel). PI, on the other hand, can enable microrobots to act independently by sensing and adapting themselves to the environment they are operating in (e.g., drug release at a certain pH level)^60^. However, despite all advances in the fabrication and actuation of microrobots, translation of these medical devices from bench to bedside is challenging yet. While the cost-effective mass production of microscale robotic devices is still a challenge to be solved, microrobots have to face obstacles from their entry into the body to the target site (e.g., being attacked and removed by the immune system of the body)^61^. In addition, current test standards to ensure the safety and functionality of microrobots require cumbersome and costly procedures, delaying the early translation of microrobots for commercialized clinical applications (Fig. 1)^62^.

In this review, the recent advancements in the actuation and fabrication of 3D-printed microrobots are reported. Furthermore, most recent end-applications of 3D-printed microrobots in environmental sectors (e.g., water treatment) and biomedical sectors (e.g., drug delivery, surgical procedures, cancer therapy, imaging, particle monitoring and tracking, sensing, and tissue regeneration) are presented in detail to provide a glimpse of what has been done so far. From a future perspective, the foreseeable applications of smart materials^63^ and AI/PI in the production of intelligent microrobots are discussed, along with current challenges towards the translation of microrobots from laboratory to clinics^64–66^.

## Methods

### Materials

Polymers, including photopolymers, photo-crosslinkable hydrogels, and natural proteins, are among the most well-known material for the fabrication of microrobots using 3D printing. Photopolymers are advantageous as they can be laser-printed into 3D objects through a photochemical reaction method^67^. Photoinitiators, monomers, and additives are the fundamental photopolymer materials used in laser 3D printing^68^. Complementary data regarding photopolymers can be found elsewhere^1,69–71^. In addition to SU-8 (an epoxy-based negative photoresist mainly composed of bisphenol^72^), IP-series photoresists (e.g., IP-S, IP-Dip, and IP-L) are a group of standard photopolymerizable substances established for high-resolution 3D microfabrication. Advantages of using IP-series included ease of handling and shape fidelity which makes them a proper substance for the fabrication of functional micro-optics and biomedical microdevices.

Hydrogels are 3D polymeric networks with a high level of hydration and have been one of the most functional types of substances for 3D-printable inks^48^. They are of significant interest for their structural similarity to the natural extracellular matrix (ECM). Hydrogels can be obtained from natural and synthesized derivatives. Examples of materials are organized in Table 1, including their properties and applications.

**Table 1:** Structures and applications of materials used in the fabrication of microrobots

Material	Structure	Application	Ref.
SU-8	Hexahedral microrobot	Multifunctional microrobots for targeted cell delivery	31
	Microrobot	A magnetic microrobot for transportation and delivery of targeted cells	288
	Swimming microrobots	Targeted transport of cargos	289
	Magnetic microrobots	On-demand cell-to-cell delivery	290
IP-L 780	Helical swimming microrobots	Targeted, single-cell, and remotely guided drug delivery	291
	Magnetic helical microswimmers	Targeted gene delivery	292
	Optically controlled microrobots	Microsurgery	157
	Magnetic microswimmers	Medical diagnosis and accurate therapy	293
IP-Dip	Magnetic microrobots	Targeted stem cell transplantation	294
	Microswimmers	Single-particle manipulation	223
	Magnetic micromotors	Targeted trapping, transportation, and discharge of motile sperms	224
GelMA	Magnetic microswimmers	Targeted delivery and discharge of theragnostic cargos	149
	Soft helical microswimmers	Diagnostics and targeted delivery	295

### 3D printing fabrication methods

**Fig. 2:**
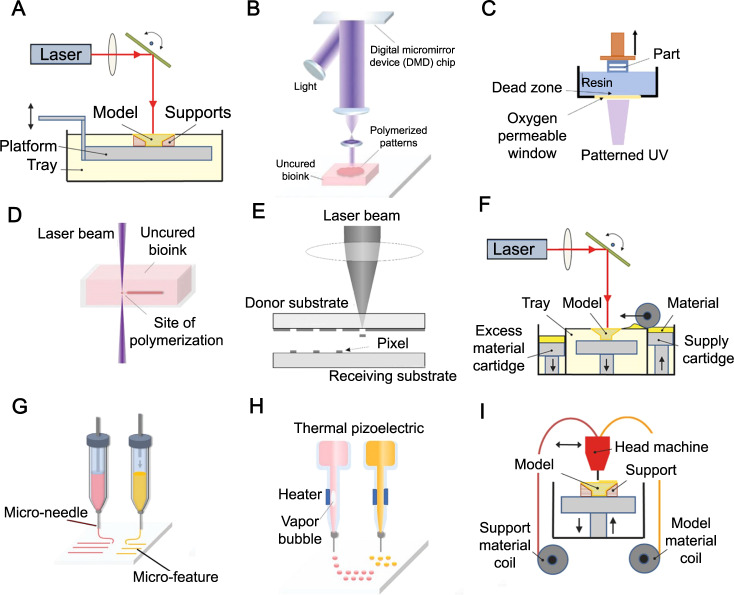
Schematics of the 3D printing technologies used for fabrication of microrobots. **A** Stereolithography (SLA). **B** Digital light processing (DLP). **C** Continuous liquid interface production (CLIP). **D** Direct laser writing (DLW) or two- or multi-photon polymerization (TPP or MPP). **E** Laser-induced forward transfer (LIFT). **F** Selective laser sintering (SLS). **G** Microextrusion 3D printing. **H** Inkjet 3D printing. **I** Fused deposition modeling (FDM). Subfigures **A**, **F**, **I** are reproduced with permission under CC BY 4.0 licenses from ref. 301; subfigures **B**, **D**, **G**, **H** are reproduced with permission from ref. 302, copyright 2020, Wiley-VCH GmbH; subfigure **C** is reproduced with permission under CC BY 4.0 licenses from ref. 303; and subfigure **E** is reproduced with permission under CC BY 4.0 licenses from ref. 298.

The 3D printing techniques used in the fabrication of microrobots (Fig. 2) are (i) stereolithography (SLA), (ii) digital light processing (DLP), (iii) continuous liquid interface production (CLIP), (iv) direct laser writing (DLW)—also known as two- or multi-photon polymerization (TPP and MPP), (v) laser-induced forward transfer (LIFT), (vi) selective laser sintering (SLS), (vii) microextrusion 3D printing, (viii) inkjet 3D printing, and (ix) fused deposition modeling (FDM). In this section, the working mechanism of each technique is briefly explained, along with a summary of the utilized materials, the offered resolution, advantages, and limitations of each method (Table 2).

**Table 2:** A summary of materials, resolution, advantages, and limitations of methods used in 3D printing of microrobots

Method	Material	Resolution	Advantages	Limitations	Ref.
Stereolithography (SLA)	Resin including photo-activated monomers—hybrid polymer—epoxides—DC 100 (high accuracy)—DC 500—DL 350/360 (high flexibility)—AB 001—GM 08 (high flexibility)— DM 210–DM 220	10 μm	Fine spatial resolution—high quality—good surface quality—good precision	Slow printing rate—limited choice of material—costly	95,296
Digital light processing (DLP)	Diamond micro particle—epoxides—acrylate-based resin—super CAST—super WAX	25-100 μm	High printing accuracy—low cost—faster printing compared to SLA—less influenced by oxygen interference as opposed to SLA—low initial vat volume is needed	Limited mechanical properties	79,81,95
Continuous liquid interface production (CLIP)	Acrylates—rigid polyurethane (RPU)—flexible polyurethane (EPU) (impact resistant)—elastomeric polyurethane—cyanate ester (CE)	75 μm	Higher production speed compared to DLP	Low-viscosity resins needed	79
Two/multi-photon polymerization (TPP/MPP)	Acrylates	100 nm	High spatial resolution	Low printing speed—restricted material choice	79,81
Laser-induced forward transfer (LIFT)	Metal substances and complexes—hydrogel materials	10 μm	Fine resolution—wide material choice—capability for printing inks in a wide range of viscosities with embedded particles	Slow fabrication process	86,297,298
Selective laser sintering (SLS)	Compact fine powder metals—alloys and limited polymers —PA12—PEEK—titanium (biocompatible)—stainless steel—aluminum—cobalt/chrome—nickel-based alloys	100–250 μm	Low anisotropy	Rough surfaces—poor reusability of unsintered powder—slow printing—lower mechanical properties due to the porous structure	79
Microextrusion 3D printing	PEG—pluronic acid—nanostructured bioinks—alginate—gelatin	150 μm	Capability to utilize high viscosity bioink and print with high cell density	Distortion of cell structure is conceivable	299,300
Inkjet 3D printing	Inks or pastes containing a concentrated dispersion of particles in a liquid	50–200 μm	Fast printing	Low adhesion between layers	81,296
Fused deposition modeling (FDM)	Thermoplastic polymers—fiber-reinforced polymers— PLA—ABS—ASA—Nylon 12—PC—PPSF/PPSU—PEI or ULTEM (Biocompatible)—PLA—TPU	50–200 μm	Low cost—high speed—simplicity	Weak mechanical properties —limited material (thermoplastics)—layer-by-layer finish—rough surface—high temperature during the extrusion process (incompatible for cells)	81

#### Stereolithography (SLA)

The SLA setup (Fig. 2a) includes a tank containing photocurable liquid photo resins (e.g., monomers and polymers such as polyglycerol sebacate acrylate (PGSA)^73^), an ultraviolet (UV) laser source provoking liquid resin crosslinking (i.e., photopolymerization), a system enabling laser beam horizontal movement, and a system directing the manufacturing platform’s perpendicular movement^74^. The manufacturing platform can move layer-by-layer vertically, following curing each resin layer to create a solid 3D structure^75^. Due to the resolution limitations of SLA, relatively high cost, limited material choice, and slow printing rate, it is not widely in use for submicron microrobot fabrication. Instead, SLA can create microscale molds rapidly and, therefore, enable the mold template-assisted 3D microfabrication of functional microrobots by incorporating other construction methods^1^.

#### Digital light processing (DLP)

Both DLP and SLA methods use light to selectively cross-link a photo resin, layer-by-layer, to create a free-standing structure. However, in DLP, unlike SLA, each layer is not exposed point-by-point but instead all at once using a selectively masked light source (Fig. 2b)^76^. There is an analogy between DLP and classical lithography and is frequently associated with dynamic mask photolithography. The data for every layer of the construction is given in the form of black and white images, which is exposed through thousands of individually adjustable digital micromirror devices (DMD)^77^. Thus, the construction time of DLP is significantly less than SLA owing to the layer-at-once (slice) exposure. Moreover, DLP is less influenced by oxygen interference (compared to the SLA) since the resin layer being polymerized is immersed in the vat and not in direct contact with ambient air. However, limited material choice and low-resolution range (~25-100 μm, which is not favorable for microrobots with complex geometrical features) are the main pitfalls of DLP. Diamond microparticles, acrylate-based resin, super CAST, and super WAX are the typical material used in DLP^78,79^.

#### Continuous liquid interface production (CLIP)

CLIP (Fig. 2c) utilizes an oxygen-permeable film to inhibit polymerization at the surface close to the UV source, eliminating the need for an intermediate recoating step for each layer^80^. Both CLIP and DLP use almost the same polymerization method, while CLIP has a UV and oxygen-permeable window at the base of the vat instead of the tiltable stage. In addition, CLIP has a higher printing speed compared to the DLP technology, enabling the generation of objects with a z-axis growth rate of 30 cm/h^79^. However, the need for low-viscosity resin and relatively limited resolution (for microrobot fabrication) are drawbacks of CLIP. Acrylates, rigid polyurethane (RPU), flexible polyurethane (EPU) (impact resistant), elastomeric polyurethane, and cyanate ester (CE) are the most used material in the CLIP process^80,81^.

#### Two- or multi-photon polymerization (TPP/MPP)

DLW (Fig. 2d), also known as TPP/MPP, enables 3D printing with sub-100-nm resolutions^82^ as a result of the nonlinear absorption of two or more photons by photosensitive monomers^83^. TPP/MPP focuses a femtosecond laser beam (near-infrared (NIR)) within a diminutive volume (i.e., voxel, 3D counterpart of two-dimensional (2D) pixel in photography) inside a photosensitive resin vat. DLW offers uniform finishes and high resolution (relatively the best resolution among currently available 3D printing techniques), whereas the point-by-point nature of TPP/MPP slows down the printing process^84^. Acrylates are the most used material for this process^81^.

#### Laser-induced forward transfer (LIFT)

LIFT (Fig. 2e) consists of three main parts: (i) a pulsed laser (to concentrate on the slim ink layer), (ii) a laser-transparent donor substrate containing the layers of the ink, and (iii) an acceptor substrate^85^. Accordingly, the computer-controlled movement of the manufacturing stage or scanning mirror can create convoluted 2D or 3D structures^86^. LIFT allows drop-based deposition of a wide variety of metal substances such as Au, Cu^87^, Al^88^, Pt^89^, metal complexes (e.g., SnO_2_ precursor^90^), and hydrogel materials^91^. Although LIFT is a timely process, high resolution, wide material choice, and the capability to print particle-embedded inks in a wide range of viscosities are the main advantages offered^85^.

#### Selective laser sintering (SLS)

SLS (Fig. 2f) is the selective heat-based sintering of a powder pool using a laser beam, causing adjacent powder particles to join together by molecular diffusion, followed by a dispensation of the subsequent powder layer^92^. The characteristic resolution of SLS is defined by powder particle size, laser intensity, and laser scanning interspace/pace^93^. Relatively low printing resolution, low mechanical strength (porous structure), rough surface quality, the need for a high power supply, high printing temperature (which can be detrimental to biomaterial being printed), and limited material choice are the main challenges associated with SLS^81^. Nonetheless, low anisotropy is the main advantage of the method^94^. Polycaprolactone (PCL), polyamide (PA), and compact fine powder metals/alloys are generally used materials for SLS 3D printing^95^).

#### Microextrusion

Microextrusion 3D printing (Fig. 2g) represents an additive manufacturing process where components are made from the bottom-up to produce 3D characteristics, and the resulting characteristic has one of its dimensions in the micrometer scale at least^96^. Material is dispensed and controlled by mechanical (e.g., piston, screw, or pneumatic), electric (e.g., piezoelectric), and/or acoustic actuators connected to a computer system. It normally delivers consecutive filaments and hence is differentiated from other droplet-based digital writing technologies, such as inkjet and electrohydrodynamic jet (e-jet) printing. Being maskless, cost-effective, the capability to print high cell density, and a wide range of material/viscosity choices are the main advantages. On the other hand, distortion of cell structure and cell damage due to the stresses experienced by cells during the printing process are limitations of this method. Polyethylene glycol (PEG), pluronic acid, nanostructured bioinks, alginate, and gelatin are used widely material in this method^97^.

#### Inkjet based

Inkjet 3D printing (Fig. 2h) operates by receiving digitalized data from the computer and generating physical objects on substrates by ink droplets^98^, hiring a drop-on-demand (DOD) jetting system, in which the actuator produces pulses, resulting in the ejection of a single droplet^99^ through a piezoelectric or thermal print head^100^. In addition, a continuous inkjet (CIJ) system can be utilized, in which ink supplies are repeatedly seized out under pressure to produce a jet that breaks up into charged droplets with steered motion with an electrical field^101^. This method not only can print polymeric materials^102^, but it also enables the printing of computer data onto substrates such as papers or transparencies^103^. Inkjet printing can employ catalytic enzymes and biocompatible inks to build microscale blocks, accordingly having manifested the capability to produce chemical-physical powered micromachines, including biocompatible microstirrers^104^ and microrockets^1,105^. However, despite being a fast printing method, low adhesion between printed layers should be considered.

#### Fused deposition modeling (FDM)

FDM (Fig. 2i) is a technique to fabric 3D structures by melting thermoplastic materials and extruding them through a nozzle^79^. FDM allows the straightforward construction of complicated 3D shapes with lower costs (compared to previous methods). Moreover, the fabrication of structures with the combination of two or more materials can be achieved using FDM (i.e., multi-nozzle FDM)^106^. However, lower resolutions compared to lithography techniques, high printing temperature (i.e., low biocompatibility), limited material choice (thermoplastic material), and layer-by-layer finish are the main trade-offs of FDM^107^. Suitable materials for FDM include thermoplastic elastomers, polylactic acid (PLA), acrylonitrile butadiene styrene (ABS), acrylonitrile styrene acrylate (ASA), Nylon 12, PC, polyphenylsulfone (PPSF/PPSU), polyetherimide (PEI), and thermoplastic polyurethane (TPU)^81,108,109^. TPU filaments are able to elongate up to 700%^110^, which can be used for the production of stretchable electronics and soft robotics, where high elongation is preferred^111^.

### Applications

According to Oxford English Dictionary, a “robot” is a machine capable of performing complicated series of tasks automatically^112^. As the name suggests, “microrobots” refers to any type of robot of submillimeter size^1^. Microswimmers are a subdivision of microrobots with the ability to move in liquids (e.g., bodily fluids or water) autonomously or under external actuation^113^. Nonetheless, the term “microswimmers” have been in use for natural micron-sized swimmers (e.g., bacteria, sperms, archaea, and protists), even before the invention of microrobots^114–116^. Therefore, neither “microrobots” nor “microswimmers” can be considered a full subset of the other one – not all microrobots are microswimmers and vice versa^116^. In what follows, based on the term used in the original works covered herein, the terms “microrobot” and “microswimmer” are used interchangeably as microscopic-scale devices that are designed to move using natural drivers (e.g., bacteria) or in response to external stimuli. Other commonly used nomenclature for microrobots can be based on their shape (e.g., microtube^117^ and microhelix^118^), driving force (e.g., spermbot^119^ and bacteriabot^120^), or behavior (e.g., microroller^121^).

3D-printed microrobots, as an emerging field of study, have found applications in environmental (e.g., water treatment), disease therapy (e.g., targeted drug delivery and microsurgery), tissue regeneration, and imaging. In this section, a review of the most recent end-applications of 3D-printed microrobots is presented. Table 3 summarizes the key advantages/capabilities and limitations of microrobots presented in this section.

**Table 3:** A summary of reported 3D-printed microrobots for different applications along with their key advantages/capabilities and limitations

Application	Capabilities and advantages	Limitations and challenges	Ref.
**Environmental applications**			
Water treatment	– Capability to detect and eliminate heavy metal ions, decontaminate organic pollutions, absorb oil droplets, and detoxify dangerous bacteria, viruses, or protozoa – Sunlight-actuated decontaminator microrobots can be distributed in water resources with no need for an external power source (possible future application)	– Limitation in traveling long distances in the range of tens of meters using current actuation methods – The difficulty of recollection and recycling after being distributed in large water reservoirs	127–131,134
**Biomedical applications**			
Drug delivery	– Ability to release drugs at targeted sites with specific environmental properties (e.g., a particular pH) or upon receiving external stimuli (e.g., specific light intensity, magnetic or acoustic pulse) – The ability for controlled/selective degradation upon completing the delivery task – Microrobot swarms can be used for large-scale drug delivery	– Long degradation hours or partial degradation (possible hazardous remnants) – Inadequate drug release performance based on the limitations of actuation methods (e.g., the restricted penetration depth of light in the skin to provoke release procedure) – Unexpected changes in environmental parameters (e.g., pH) can result in misguided drug release at undesired sites	139–142
Surgical procedures	– Optical manipulation capability (e.g., optical tweezers as a precise method) – Ability to fabricate four-dimensional (4D) microstructures (by typical 3D printers) using smart materials that can be programmed to respond to a specific stimulus (e.g., temperature, pH, or magnetic field)	– Potential cell damages in light-based actuation methods – Possibility of lower biocompatibility of microrobots as a result of adding functional groups to the microrobots (e.g., smart material) – Possible malfunction of delicate moving components owing to the unpredicted environmental changes (e.g., pH and temperature) – Changes in surface polarity can unfavorably change the swimming speed of the microrobot	151,157,158
Particle monitoring/tracking and imaging	– The capability of in vivo imaging and real-time tracking – Imaging can be done by currently in use magnetic resonance imaging (MRI) devices – Monitoring accuracy can be improved by coloring microrobots without the need for complex functionalization steps	– Microrobots may be displayed as a cloud (not distinguishable individually) due to restricted spatial resolution – Limitations in trackability of particles based on the operational range of the imaging system (e.g., multispectral optoacoustic tomography (MSOT))	160–163
Sensors and actuators	– Microrobots can be integrated with different sensing mechanisms, such as an optical force sensor – Facilitating quick and precise micromanipulation – The capability of operating with on-board and off-board actuation methods	– Possible toxicity by using metallic parts – The possibility of inaccurate readouts as microsensors are sensitive to subtle changes in environmental parameters (e.g., temperature) – Limitations in the functionality of the microrobots based on the specific working range of the actuation methods	166,169,175
Tissue regeneration	– The ability to deliver stem cells to the target tissues inside the body – Sensitive delivery without sacrificing the stemness capability of cells	– Perilous spread of pathogenic cells in the body in case of malfunction of the microrobots – The biocompatibility of microrobots may decrease by functionalizing the surface of the microrobots – Potential barrier by the fibrous elements of the tissue extracellular matrix (ECM) for the functionality of the microrobots	158,174

#### Environmental applications

Adverse environmental repercussions of industrialization and the surge of the population have attracted attention to remediation approaches^122–126^, such as detection and elimination of heavy metal ions^127^, reduction of organic pollutions, and adsorption of oil droplets^128–130^. To perform actual environmental missions, traveling long distances in the range of tens of meters is important for microrobots. For this purpose, a 3D-printed millimeter-scale motor (3DP-motor) was proposed, which acted as an “aircraft carrier” of TiO_2_/Pt Janus micromotors to assist environmental remediation applications in large volumes^131^. An FDM 3D printer was used to manufacture the motors with PLA filaments with a diameter of 1.75 mm. The propulsion of the 3DP-motor was achieved based on the Marangoni effect by generating a surface tension gradient with asymmetric releasing of ethanol from the 3DP-motor tank. Simultaneously, a gradual discharge of the TiO_2_/Pt Janus micromotors could promote their quick distribution and degradation action over a vast area. For activating the Janus particles towards the degradation of 2,4,6-trinitrophenol (TNP), a broad wavelength Xenon lamp was hired to simulate sunlight. Maximum velocity, average velocity, lifetime, and traveled distance of 70 m/s, 17.9 ± 6.8 µm/s, 17–21 min, and 30 min were achieved, respectively, for the optimum conditions^131^.

#### Water treatment

Water pollution endangers public healthcare globally, as polluted water is the birthplace of many illnesses^132^. Untethered microdevices^31,133^ have been introduced as possible movable environmental micro cleaners for detoxification of dangerous bacteria, viruses, or protozoa and elimination of toxic chemicals, having the ability to swim in difficult-to-access areas (e.g., pipes or conductions)^134^. A shortcoming of these devices is the fact that most of them cannot be reused. In this regard, a microrobotic prototype with the aim of water cleaning was fabricated by combining the wet metallization approach and SLA 3D printing technology, which is suitable for the cheap realization of millimetric-sized microrobots with complex structures^135^. Metallic layers (silver and titania) were deposited to enable bacteria-killing and photodegradation features. In addition, a magnetic layer made of a cobalt-nickel phosphorous (CoNiP) alloy, was used to navigate microrobots remotely. Silver/titania coated microrobots eliminated 89% of bacteria in the specimen, compared to 38% bacteria-killing performance for uncoated 3D-printed units^135^.

In another study, smart-dust robots were fabricated with FDM 3D printing for autonomous motion (i.e., magnetic responses) using graphene filled with aluminum/gallium molten alloy (Al/Ga)^136^. Owing to their outer surface coated with a hydrogel/photocatalyst layer consisting of chitosan, carbon nitride, and C_3_N_4_, the robots were able to swim by reacting to the medium they were placed in—water, without a need for fuel addition, making these robots, so-called, eco-friendly robots. The primary application of the robot was the photocatalytic degradation of the picric acid as an explosive model molecule under visible light. A single robot consisted of a hollow tube with 1 mm inlet and 2 mm outlet diameters, and 8 mm length. A higher ratio of Al compared to Ga led to faster swimming velocities. The swimming speeds varied between 60 µm/s, for 1:5, to 200 µm/s, for 1:1 ratios^136^.

#### Biomedical applications

Being on a microscale enables microrobots to be moved remotely in hard-to-reach sites in the human body to perform therapeutical duties. A process to manufacture iron microrobots was presented using template-assisted electrodeposition inside TPP 3D-printed micromolds^137^. The produced Fe-microrollers and microswimmers had a movement speed of ~20 body lengths per second. The five-turn helix with the radius and pitch distance of 25 and 20 µm, respectively, was capable of swimming at the maximum average velocity of 42 µm/s while being operated in 100 cSt silicone oil at 10 mT and 7 Hz^137^.

The immune system has a defense mechanism to neutralize foreign particles, such as microrobots. Immunological macrophages recognize foreign particles and attack them through a process called phagocytosis, which results in the demolition of microrobots. A 3D-printable zwitterionic photoresist was utilized to produce microrobots that can avoid phagocytosis^138^. Zwitterionic photoresists eliminated the toxicity brought about by PEG-based microrobots since zwitterionic was produced with water-soluble photoinitiators as opposed to organic solvents used with conventional materials. The ability of zwitterionic to avoid immunological attacks was proven by subjecting the 3D-printed microrobots to immune cells, including T-lymphocytes, B-lymphocytes, dendritic cells, and macrophages. More than 98% of the zwitterionic microrobots evaded any interaction with immune cells, while almost all of the PEG-based microrobots, with the same shape were captured by immune cells^138^.

##### Drug delivery

**Fig. 3:**
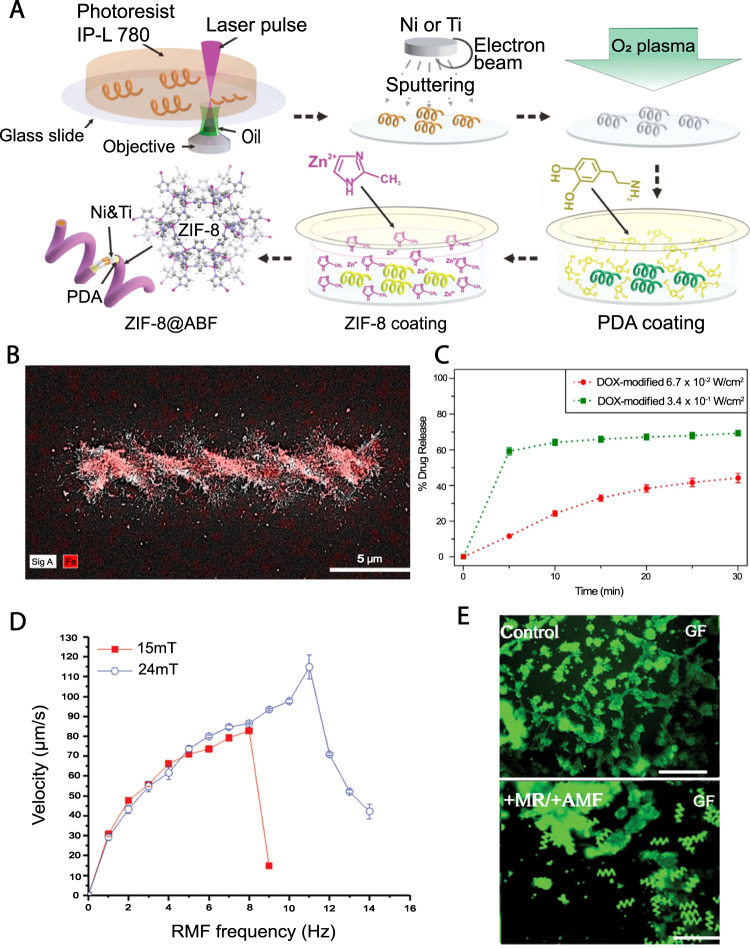
3D printed microrobots for drug delivery. **A** ZIF-8 coated, biocompatible, and pH-responsive drug carrier helical microrobots. Graphic representation of the production steps of ZIF-8@ABF microrobots. Reproduced with permission from ref. 139. Copyright 2019, WILEY-VCH Verlag GmbH & Co. KGaA, Weinheim. **B**, **C** Three-dimensional (3D) printed, magnetically actuated chitosan microswimmers with the ability to release drugs with light excitation. **B** Energy-dispersive X-ray spectroscopy elemental mapping showing the presence of iron atoms (red color) in the microswimmers. **C** Total DOX release from the microswimmers with time for 6.7 × 10^−2^ and 3.4 × 10^−1^ W/cm^2^ light intensity. Higher light intensity was directly correlated to the release rate^140^. Subfigures **B** and **C** are reproduced with permission from Bozuyuk, U. et al. Light-triggered drug release from 3D-printed magnetic chitosan microswimmers. ACS Nano 12, 9617-9625 (2018). Copyright 2018, American Chemical Society. **D**, **E** Characterization of the magnetic propulsion and drug encapsulation capabilities of biodegradable hyperthermia microrobots (DHM). **D** Velocity of microrobots in respect to RMF frequency. The “step-out” (i.e., the sudden plummet of swimming speed) happened in lower frequencies/speed for 15 mT RMF. Hence, in order to acquire higher speeds, higher FMF frequencies are required. **E** Green-fluorescence images of in vitro test of the hyperthermic effect of DHMs on HCT116 cells, confirming the potency of DHMs for targeted hyperthermia therapy. Control: HCT116 cells only; +MR/ + AMF: DHM with AMF. Scale bars are 200 µm. Subfigures **D** and **E** are reproduced with permission from ref. 150. Copyright 2019, WILEY-VCH Verlag GmbH & Co. KGaA, Weinheim.

Helical micromachines were developed based on a metal−organic framework (MOF) and actuated via weak magnetic fields, named artificial bacterial flagella (ABF), with the ability to release drugs at targeted mediums with specific pH values (Fig. 3A)^139^. ABF structures were made using TPP 3D printing of IP-L 780 photoresist, followed by a Zeolitic imidazole framework-8 (ZIF-8) coating. This nickel- and titanium-based coating not only allows magnetizability and biocompatibility, but also enables selective degradation in mildly acidic environments such as tumor regions (pH ≈ 6) owing to its zinc-based outer layer. The RhB-ZIF-8-ABF composite structure remained stable for 24 h inside the PBS solution, which had a pH value of ~7.4, where rapid decomposition occurred in a solution with a pH of 6^139^.

A magnetic microswimmer was produced for light-induced drug delivery (Fig. 3B, C)^140^. The swimmers with dimensions of 6 μm in diameter and 20 μm in length, were fabricated by TPP using photo-cross-linkable polymer methacrylamide chitosan (ChMA) embedded with SPIONs. Printing of an individual microrobot was performed in 10 s, and the fabricated device was successfully maneuvered inside the water with an average speed of 3.34 ± 0.71 μm/s under a 4.5 Hz and 10 mT rotating magnetic field. Photocleavable linker molecules broke into two components upon exposure to a 365 nm wavelength light, releasing the drug from the composite. It was shown that virtually 60% of the microswimmer-linked drug was unbound after being exposed for 5 min. The degradability of microswimmers was tested by resolving them with lysozyme enzyme, which demonstrated a partial degradation after 204 h^140^.

Comprehending biological processes, such as cell–cell interactions and cellular response to mechanical stimuli, is still a challenge due to the technological limitations associated with the manipulation of single cells in biological liquids. Although optically trapped microbead technology is currently one of the most used methods for single-cell analysis, it offers limited design options, and its strong lasers lower the biocompatibility of this system^141^. 3D optical manipulation of microrobots in real-time was implemented using a fluorescent photocurable resin that contains quantum dots (Qdot)—to label cell trapping regions of microrobots, while the rest of the robot was made of a non-fluorescent resin^141^. Hybrid 2-photon micro SLA 3D printing enabled the process of the resin with a resolution of ~100 nm. The modified resin was able to keep attached cells for a more extended period of time (on the scale of minutes), unlike non-modified resin that was not able to keep cells for a prolonged duration. A microscope was used, which was able to render 1 µm trap points of the microrobot^141^.

Nanoscale 3D printing was employed to build self-propelled microscopic tori–ring-shaped structures, steered by an external magnetic field^142^. The tori, with a diameter of 3 and 7 μm and thickness of ~750 μm, were made by TPP 3D printing using an IP-DIP photoresist followed by a coating of nickel and platinum through thermal evaporation. The catalytically active platinum layer enabled self-swimming of the tori across the surface of a gold-coated glass slide at a velocity of 7 μm/s in the presence of 30% hydrogen peroxide. The microstructures were used to carry positively charged 2 μm latex spheres and 1 μm polystyrene beads on their cathodes and anodes, respectively, which can be released by a magnetic pulse in the desired sites^142^.

The pathogenesis of neurodegenerative dysfunctions, such as Parkinson’s as well as Alzheimer’s diseases and traumatic injuries of the central nervous system, ordinarily results in irreversible neuronal injury and neuronal death^143^. Unlike traditional medical remedies, e.g., gene therapy and neurosurgery medications, cell treatment exhibits great prospective for repairing the damage to the central nervous system tissue^144^. In this regard, helical-shaped TPP 3D-printed soft magnetoelectric microswimmers (100 µm long) were produced to transfer and differentiate neuron-like cells^145^. Multiferroic nanoparticles, presenting magnetoelectric characteristics (MENPs), could be considered as a motile element under rotating magnetic areas and as electric­field nanogenerators during subjection to alternating magnetic fields. This gave the microswimmer the ability to utilize a single energy source for both navigation and trigger of its stimulating function, ultimately simplifying the design and control process^145^.

A magnetically actuated (using rotating magnetic fields (RMFs)), helical, porous, and degradable microrobot (PDM) was produced by TPP of poly (ethylene glycol) diacrylate (PEGDA)–pentaerythritol triacrylate (PETA), including anticancer drug 5-fluorouracil (5-FU) and magnetite nanoparticles (Fe_3_O_4_), with a diameter and length of 40 and 120 µm, respectively^146^. The use of porous PDM increased surface area, which in return caused a higher response to the acoustic energy, resulting in the promotion of the acoustically provoked drug release. Despite the possible damage to cells by acoustic pulses (which can be partially alleviated by optimization of the exposure time and intensity of the ultrasound pulses^147^), acoustic fields were able to be accurately localized on a certain location using a focused ultrasonic beam^148^. Therefore, the acoustic field intensity of 1 W/cm^2^ (with 40 min exposure time) was found as the maximum ultrasonic stimulation condition with no severe adverse influence on cells. The difference in the quantity of discharged 5-FU in the first hour was 66% greater for the PDM group compared with the NPDM group, indicating that the porous surface promoted ultrasound-induced drug discharge^146^.

Cancer cells use the matrix metalloproteinase 2 (MMP-2) enzyme in the metastasis process to escape from the surrounding matrix, elevating the local concentration of MMP-2. A theragnostic method (a combination of therapeutics and diagnostics) was introduced using TPP 3D printing of a hydrogel-based, biodegradable, microswimmer based on the environmental sensing of MMP- 2 enzyme to perform theragnostic load (e.g., medicine) transportation and targeted discharge (double-helical architecture with length and diameter of 20 and 6 μm, respectively)^149^. It was reported that the MMP-2 enzyme could fully degrade the microswimmer in 118 h to solubilized nontoxic products, for 0.125 μg/mL concentration, while the required time for 0.500 and 4 μg/mL concentrations were 67 and 5 h, respectively^149^.

Helical, degradable hyperthermia microrobots (DHM), which facilitate magnetically actuated drug delivery and hyperthermia therapy, were created with TPP 3D printing (Fig. 3D, E)^150^. DHMs with a diameter of 40 µm and length of 120 µm were made out of PEGDA–PETA composition that contains Fe_3_O_4_ magnetic nanoparticles (as a magnetizable element) and 5-fluorouracil (5-FU) as a cancer drug. Local heating of cancer cells was reported to be therapeutic since cancer cells are more vulnerable to heat than healthy cells. The heating capacity of the microrobots, in an AMF, was verified with a 3D-printed 5 × 1 mm PEGDA–PETA/Fe_3_O_4_ disk. Excitation of the disk with an AMF of 45 kA/m at 430 kHz increased the temperature of a 100 µl PBS solution from 28 to 40 °C in 10 min. On the other hand, the drug distribution capability of the DHMs was tested in which 90% of the attached drug was observed to dissolve after 168 h. In addition, tumor treatment efficacy of the drug carriers was tested, reporting that the cell viability was reduced to 54%^150^.

##### Surgical procedures

Optical tweezers (OTs) (also known as single-beam gradient force trap), in their simplest form, are instruments based on a highly focused laser beam, enabling optical manipulation and trapping of a wide range of microscopic and sub-microscopic objects (e.g., nanoparticles) in its focal spot^151^. OTs have recently been widely used in biomedical research approaches, including movement, stiffness measurement, classification, assembly, and cut of biological cells, as OTs are dynamically flexible and precisely maneuverable in a restricted area^152–154^. Nevertheless, cells could be harmed by being exposed to a direct light beam (i.e., local heating and/or photodamage)^155,156^. Accordingly, indirect administration of target biological particles by catching a biocompatible micro-object before utilizing it as an end effector of OTs can mitigate the light-induced damage^155,156^. In this regard, TPP was used to fabricate an articulated microrobot for indirect manipulation of cellular structures under laser light^157^. Comparing the stiffness of one-point and two-point traps, it was shown that the stiffness in the Y direction was 15 times higher and for the X direction was four times higher for two-point traps compared to the one-point trap^157^.

Biodegradable gelatin-based ABFs were manufactured using DLW by functionalization of gelatin (to be photocurable) with acrylic groups^158^. However, introducing functional groups (e.g., acrylates) notably lowers biocompatibility. Accordingly, an indirect method to manufacture 3D-printed sacrificial templates (high-resolution micromolds fabricated by DLW) was proposed. Besides, the developed method led to the fabrication of four-dimensional (4D) stent-like microstructures with a minimum dimension of 5 µm. NOA63, as a shape memory polymer (SMP), was used in the 4D printing section of this study. SMPs were not manufactured using DLW since obtaining the appropriate combination of photoinitiators and monomers to achieve biocompatible SMPs, with operation temperatures in the range of body temperature, is still challenging. While Young’s modulus (E) for the recorded 4D printed stent is about 100 MPa^159^, the E of the designed microstent was in the order of 1 GPa, similar to those of commercial polymeric medical stents^158^.

##### Particle monitoring/tracking and imaging

Magnetically actuated helical micromachines (ABF with a length of 8 and 16 µm, coated with Ni and Ti) were produced by an integration of DLW and TPP for fluorescent imaging applications in vivo (imaging of peritoneal cavity of a mouse)^160^. The swimming capability of the ABFs was tested in vitro in 5% dextrose solution, and an average speed of 70.4 µm/s was recorded in a 9 mT, 90 Hz magnetic field. In order to demonstrate the in vivo imaging ability of the micromachines, a flock of fluorescently labeled ABFs was inserted into the intra-peritoneal cavity of a 4-week-old Balb/C mouse, which appeared as a cloud under an in vitro imaging system (IVIS). The cloud was effectively moved ~1.25 mm in 5 min (average velocity of 6.8 µm/s) under a 9 mT, 90 Hz rotating magnetic field^160^.

SLA 3D printing was combined with electroless metallization to create resin-based (DL260), magnetically movable scaffolds (with lengths of 4.5 and 3 mm) with cell carrying ability^161^. In order to examine the movability, the scaffolds were immersed in water and oil, and exposed to RMFs. The linear speed of scaffolds increased by an increase in magnetic field frequency in water and air, while the speed was limited to roughly 1 mm/s (for frequencies above 0.8 Hz) in oil. Moreover, the effect of the scaffolds on cell viability was inspected, showing 90% cell viability for cells that were exposed to gold-coated scaffolds after 2 days^161^.

Real-time tracking of TPP 3D-printed micro-objects under a tissue imitating media was performed with multispectral optoacoustic tomography (MSOT)^162^. Micro-objects with 25, 50, and 100 μm in length and diameters of 12 and 6 μm were placed under 10-mm-thick layers of PDMS- glycerol composite and agarose, containing soya milk, which had similar mechanical properties to biological tissues. Although 25 and 50 μm particles could not be detected separately because of the restricted spatial resolution of the detector, 100 μm particles were differentiated from smaller particles. In order to demonstrate the tracking ability of the microdevices, helical and cylindrical microstructures were moved inside the agarose, containing gel that had a microfluidic channel in it. Application of a ~60 mT direct current (DC) magnetic field on 100 μm microparticles moved them with a speed of 1160 μm/s inside the channel with a tracking precision of 100 µm^162^.

**Fig. 4:**
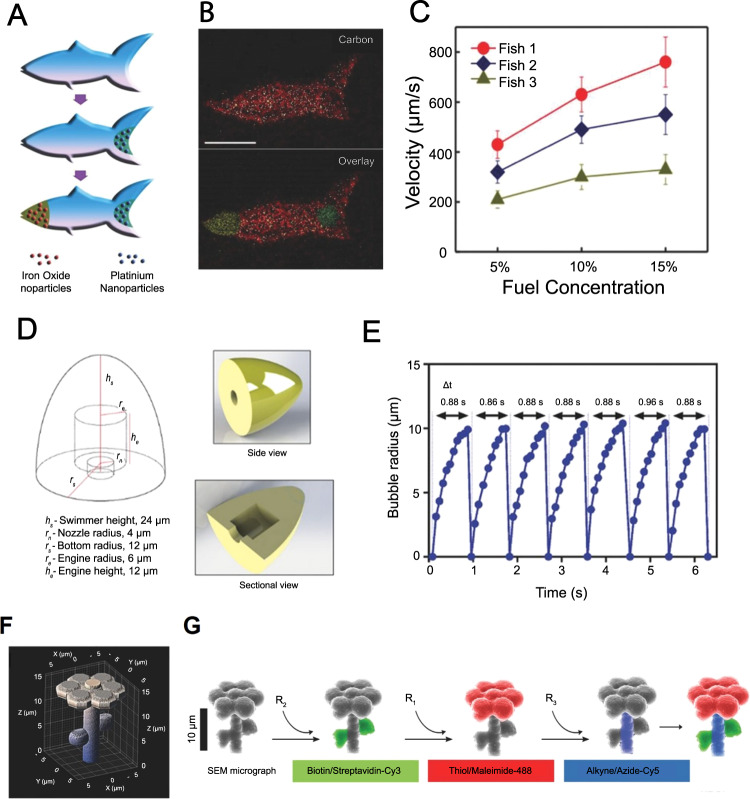
3D printing of more complex microrobots. **A**–**C** Three-dimensional (3D) printed artificial fish propelled with catalytic impulsion. **A** Functionalization of microfishes. Platinum (Pt) nanoparticles were loaded to the tail of the fish using catalytic decomposition for propulsion. Subsequently, to enable magnetic actuation/control, Fe3O4 nanoparticles were loaded on the head of the fish. **B** Energy-dispersive X-ray (EDX) spectroscopy images of poly (ethylene glycol) diacrylate (PEGDA) microfish body, platinum tail, and the iron-oxide head (scale bar: 50 µm). **C** Velocity plots of microfishes with different shapes and Pt nanoparticle concentrations (5, 10, and 15% H_2_O_2_). Fish 1: common fish with 8.0 × 108 Pt particles per ml; Fish 2: manta ray with 8.0 × 108 Pt particles per mL; Fish 3: common fish with 4.0 × 108 Pt particles per mL. Subfigures **A**–**C** are reproduced with permission from ref. 163. Copyright 2015, WILEY-VCH Verlag GmbH & Co. KGaA, Weinheim. **D**–**G** Three-dimensional (3D) printed self-propulsive microswimmers. **D** Computer-aided design (CAD) and dimensions of a low-drag bullet-shaped microswimmer with an inner cavity to produce catalytic jet bubbles. **E** Microbubble generation from microswimmer’s inner cavity at a steady frequency, demonstrating continuous/steady self-propulsion ability of the swimmers. **F** CAD and dimensions of microflowers. **G** Scanning electron microscope (SEM)-image validation of separately patterned chemical sites on microprinted flowers by fluorescent dye-coupled particles that orthogonally reacted with their target sites. Subfigures **A**–**C** are reproduced with permission from ref. 165. Copyright 2016, WILEY-VCH Verlag GmbH & Co. KGaA, Weinheim.

Synthetic microfishes that could be magnetically or chemically actuated were created (with 1 µm resolution) with a custom-designed 3D printing method called microscale continuous optical printing (µCOP) (Fig. 4A–C)^163^. Microswimmers with a thickness and length of 30 and 120 µm, respectively, were made of PEGDA by a modified DMD, which modulated UV light. Catalytic platinum nanoparticles (embedded at the back of the printed fish) enabled chemical propulsion of the microfish with a velocity of 780 µm/s in the presence of hydrogen peroxide. On the other hand, iron-oxide nanoparticles (embedded at the front) enabled the magnetic movement of the fishes in the presence of a magnetic field. In order to demonstrate the toxicity detection capability of the printed microswimmers, toxin-neutralizing 10,12-pentacosadiynoic acid (PCDA) was integrated into PEGDA. The resulting surface structure emitted a fluorescent signal upon reaction with toxins, indicating the chemical presence and disruption of toxins^163^.

Although coloring of 3D-printed microrobots can be used for tracking purposes, current methods mostly involve complex functionalization steps, and colors decay over time. In this regard, helical magnetic microswimmers, with integrated rectangular color emitting platforms, were fabricated with TPP 3D printing of IP-DIP photoresist, which enabled instantaneous identification, tracking, and closed-loop control of microrobots^164^. Different colors were achieved by forming arrays of 1 μm (or less) prisms with geometry-specific color expressions on the surfaces of microrobots. A germanium layer was grown to increase color expression. Microswimmers with red (R), green (G), or alternating red and green (RG) blocks, were exposed to a rotating 5 Hz, 20 mT magnetic field in a 0.5 cm thick silicone oil medium. Comparing colored microrobots to control microrobots (with no prism sequences), a red color expression was reported upon exposure to a high-intensity white light bulb, enabling real-time tracking and control of microrobots with a color-based algorithm^164^.

TPP was employed to create PEGDA-based bullet- and flower-shaped microswimmers with chemical propulsion capability for bullet-shaped design (using platinum) (Fig. 4D–G)^165^. Specific regions of microswimmers were chemically modified (i.e., colored) into functional groups, such as carboxylic acid, alkyne, and biotin. The self-swimming ability of the bullet-shaped microswimmers was demonstrated in low concentration hydrogen peroxide solution (1–5 vol%) with a mean velocity of 27.0 ± 5.2 µm/s. On the other hand, the potential of the TPP to create microrobots with diverse embedded functional groups was shown by modifying separate regions of a printed flower structure into biotin/streptavidin-Cy3, thiol/maleimide-488, and alkyne/azide-Cy5 regions^165^.

##### Sensors and actuators

Metallic parts are challenging to be fabricated on micro-scales. Although SLS allows direct fabrication of metallic structures, the printing resolution is not suitable for microrobot production^166^. A potential way to achieve a metallic finish on a printed object is to metalize the resin’s surface post-printing, which facilitates obtaining some characteristics of metals without having a bulk metallic object. For instance, electroless plating is a metallization method that can produce uniform and thick metal layers^167^. In this regard, electroless metallization of SLA 3D-printed objects (using Co and NiFe) was reported with a focus on the production of magnetically active cantilevers and their potential application as actuator prototypes, having 9 mm length, 0.6 mm width, and 0.2 mm thickness^168^. The co-plated cantilever had less sensitivity to the distance of the magnet in terms of tip displacement compared to NiFe-plated one. Moreover, a three-times rise in the temperature enhanced the deposition rate by ~12 times^168^.

Microscale robotic grasping devices can facilitate quick and precise micromanipulation in microsurgeries. A TPP 3D-printed, tethered, compliant grasper was developed combined with an optical force sensor on the tip of an optical fiber^169^. The developed gripper, with dimensions less than 100 µm in all three axes, could be used to probe biological microstructures, including alveoli, villi, and single cells, and had the potential to be used in invasive medical tasks, such as drug delivery, microbiopsy, and microsurgery. High-dimensional spectral readings of the optical interferometry were used to train an artificial neural network (ANN) to predict the axial force applied to the gripper. Trained ANNs were shown to precisely prognosticate the force with an average error of less than 0.8 µN, equal to an error of 2.7% over the trained force span^169^.

**Fig. 5:**
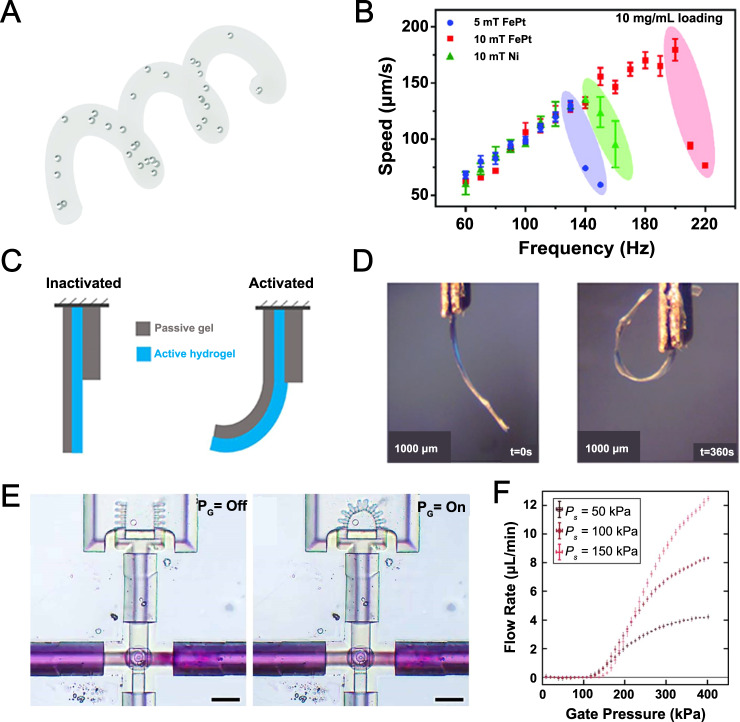
Microrobots and microactuators. **A**, **B** Two-photon-polymerization (TPP) three-dimensional (3D) printed iron platinum nanoparticle-based magnetic helical microswimmers. **A** Conceptual design of a trimethylolpropane ethoxylate triacrylate (PETA) magnetic helix with three turns and embedded FePt or superparamagnetic iron-oxide nanoparticles (SPIONs). **B** Swimming velocity of 30 μm long SPION/PETA and FePt/PETA magnetic helical microswimmers. FePt-based microswimmers were able to endure higher frequencies (with higher step-out frequency), reaching higher velocities compared to other microswimmers. Subfigures **A** and **B** are reproduced with permission under CC BY 4.0 licenses from ref. 170. **C**, **D** Three-dimensional (3D) printed soft microstructures that actuate upon water absorption. **C** Illustration of the actuation motion with hydration. The actuation was triggered by water vapor absorption. **D** The motion of a bilayer hydrogel structure during hydration with vapor for 360 s. Subfigures C and D are reproduced with permission from ref. 172. Open access, 2020, IOP Publishing Ltd. **E**, **F** Soft microrobotic grippers and microfluidic transistors fabricated by in situ direct laser writing (isDLW). **E** An integrated microfluidic system comprising of one microfluidic transistor and one soft microgripper. The grippers closed upon application of P_g_ to the transistor (right). According to the working principle of this soft microgripper, an applied input pressure (P_s_) triggered the inward movement of grippers, while microgrippers remained open when the pressure was not present. Scale bars are 50 µm. **F** Experimental results of flow rates through a 25 µm disc transistor for varying P_g_ values and P_s_ values of 50, 100, and 150 kPa. For a constant P_g_, higher flow rates were achievable using higher P_s_. Subfigures **E** and **F** are reproduced with permission from ref. 173. Copyright 2021, IOP Publishing Ltd. Reproduced with permission. All rights reserved.

TPP was utilized to create biocompatible, ferromagnetic, helical microrobots with a diameter of 3 µm and length of 30 µm using trimethylolpropane ethoxylate triacrylate (PETA) with embedded iron platinum (FePt) nanoparticles (Fig. 5A, B)^170^. Microrobots moved at a speed of ~175 μm/s at an applied magnetic field of 10 mT and 200 Hz, which was almost 500% faster than commonly used SPION integrated microswimmers and 25% faster than microswimmers coated with a 100 μm nickel layer, respectively. The FePt particles could produce more speed since FePt can be 3D printed with a density of up to 10 mg/mL in PETA, while the printing density of SPION-based PETA was limited to 5 mg/mL. The biocompatibility of the PETA-FePt device was tested with murine macrophage cells for 24 h, reporting over 90% cell viability for all concentrations after 1 day of incubation^170^.

Microscale thermal and electrostatic actuators were fabricated using TPP 3D printing (IP-S negative photoresist) and aluminum sputtering, which eliminates the need for additional steps to electrically isolate the actuators from the substrate^171^. Sidewall coverage was used to make an electrostatic actuator with interdigitated comb structures. Their deflection upon application of varying electric voltage (from 50 to 160 V) was measured with a maximum value of 12.7 μm for 160 V, demonstrating a linear relationship between displacement and voltage increment. Moreover, thermally waved, 500 μm long wing-like structures were created with the proposed method, for which a displacement of 18 μm was recorded with excitation of 8 mA. Furthermore, the wings were subjected to a cyclic test of 8 mA and 10 Hz and successfully endured 8500 cycles^171^.

Water-sensitive (actuated when subjected to water) microactuators were fabricated by extrusion 3D printing using an Ebecryl 4491 layer, as a passive layer, and Hydromed D4, as an active layer (Fig. 5C, D)^172^. A 1 mm wide, 4 mm long, and 65 µm thick actuator was made to show its ability to deform upon hydration and return to its initial condition after dehydration. The tip of the actuator was displaced for more than 5 mm upon exposure to steam for 200 s, while it returned to its initial shape after 50 s when dehydrated. In addition, the effects of the thicknesses of active and passive layers on tip displacement were examined, showing an increase in displacement for higher ratios of active to passive layers^172^.

A fluid-pressure actuated microfluidic microgrippers (with microfluidic transistors) was fabricated using IP-L 780 with a custom-designed 3D printing method named in situ DLW (isDLW) (Fig. 5E, F)^173^. The microfluidic microgrippers were designed to have two states: “open state” in the case of no input pressure, and “closed state” in the presence of pressure. On the other hand, the microfluidic transistor setup (P_g_: gate pressure and P_s_: microchannel pressure) enabled multiple flow modes inside the microfluidic device depending on pressure input. In a gate with a diameter of 25 µm, the flow was blocked under P_g_ < 100 kPa independent of the P_s_^173^.

##### Tissue regeneration

**Fig. 6:**
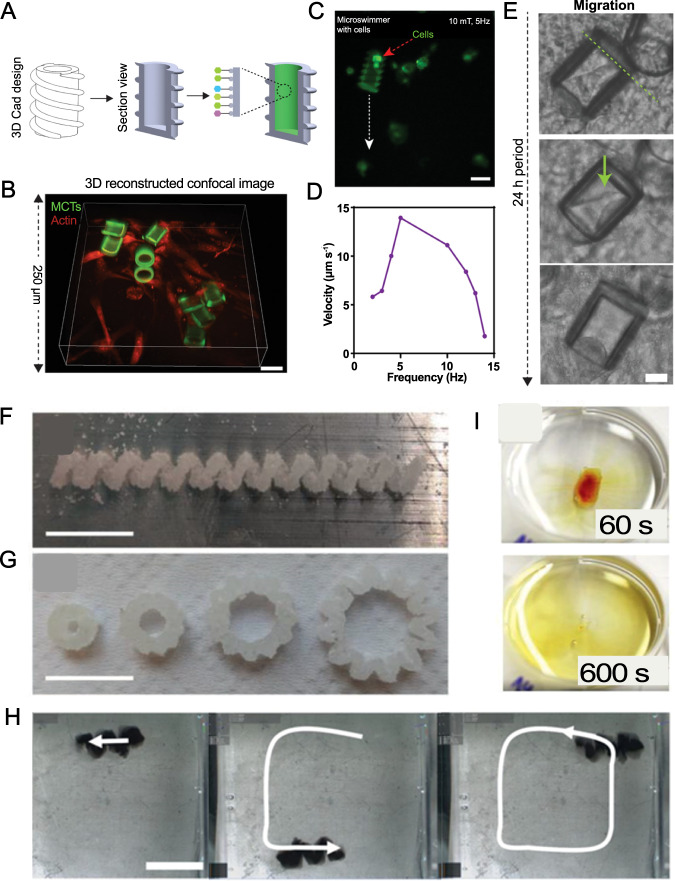
Cargo delivery and dissolvable microrobots. **A**–**E** Three-dimensional 3D-printed microrobot cell transporter (MCT) with the ability to deliver stem cells in vitro. **A** Computer-aided design (CAD) design was two-photo-polymerization (TPP) 3D-printed, and the inner surfaces of the structure were modified to increase stem cell bonding capability. **B** 3D confocal image of cell-loaded MCTs after 72 h of being in Matrigel (red: actin; green: RUNX2, osteogenic cell marker) (scale bar: 60 µm). **C** Image of an MCT with encapsulated cells during rotational actuation (propulsion) and steering in vitro with a 10 mT, 5 Hz magnetic field (scale bar: 50 µm). **D** Graph of the average speed of MCTs with respect to different frequencies of a 10 mT magnetic field. An increase in magnetic field frequency over 5 Hz resulted in a decrease in velocity since the MCT failed to convert the high-frequency rotational magnetic field to linear movement. **E** Out-migration of cells from MCT within 24 h period (scale bar: 20 µm). Subfigures **A**–**E** are reproduced with permission from ref. 174. Copyright 2019, WILEY-VCH Verlag GmbH & Co. KGaA, Weinheim. **F**–**I** Three-dimensional (3D) printing of sugar-based microrobots that can be moved magnetically. **F**, **G** Microscopy images of saccharide-based, selective laser sintering (SLS) 3D-printed structures. **F** A glucose-based helix (*P* = 22.5 W; *V* = 1.4 cm/s). **G** sucrose-based gears (*P* = 22.5 W; *V* = 1.4 cm/s). **H** Magnetic operation route of a sugar helix inside 30 wt% Water/Glycerol seen from the top. **I** Dissolution images of a sucrose-based slab in W/G at 60 and 600 s. Scale bars are 10 mm. Subfigures **F**–**I** are reproduced with permission from ref. 259. Copyright 2020, Wiley-VCH GmbH.

Stem cells are intrinsically regenerative cells that can differentiate into specific cell types depending on cell–cell and cell–matrix interactions, showing a potency for restoring damaged tissues. However, the transport of stem cells to a particular site in the body is a major challenge since stem cells could differentiate into unprompted malignant forms if mistakenly transported to a site with different properties than the site derived from ref. 174. In this regard, magnetically actuated microrobotic cell transporters (MCT) were made by TPP 3D printing with the aim of delivering stem cells to target tissues and enabling differentiation of stem cells into targeted cell lines without sacrificing the stemness capability of cells (Fig. 6A–E)^174^. A 76 µm long MCT, with a double helix outside design and a 20 µm inner cavity hole, was fabricated out of poly(ethylene glycol) and trimethylolpropane ethoxylate triacrylate (TMPETA), containing 20 mg/mL SPIONs. The surface functionalization improved cell viability from 32.2 to 77.5%. Furthermore, 86.3% of the initially attached cells retained their bonding to the functionalized surface after 3 days, whereas only 40% were stable for non-functionalized control MCTs. Embedding SPIONs into this design enabled the corkscrew motion of MCTs with a 10 mT rotating magnetic field at an average velocity of 11.14 µm/s. In order to show that conveyed stem cells can be pre-tailored to differentiate into specific cell types, MCTs were incubated with an osteogenic differentiation medium for 5 days. Runt-related transcription factor 2 (RUNX2) expression, an osteogenic marker, was increased in the cells that were grown in this medium, proving that stem cells were differentiated into osteogenic cells^174^.

### Actuation methods

Actuation of microrobots in vitro and in vivo is a challenging task considering the limitations related to their submillimeter scale. The propelling mechanism should generate enough force to overcome viscous forces (which are dominant on micron-scale, unlike inertial forces that are dominant on larger scales) without compromising the structural integrity and biocompatibility of the device^175^. The actuation methods are commonly classified as on-board and off-board methods. Microrobots with on-board actuation (i.e., self-actuated microrobots) use their preloaded chemical or biological (e.g., bacteria, sperm, and/or muscle tissue) power sources for propulsion in reaction to certain substances in their operating environment, without the presence of external actuation^63,176–178^. On the other hand, off-board methods use external force fields, such as magnetic, acoustic, ultrasound, light-driven, and/or electric propulsion, to drive microrobots in fluidic environments.

#### On-board actuation

##### Chemical actuation

Chemical self-actuation is the generation of a force by a chemical reaction of on-board molecules such as platinum^179,180^, silver^181^, iron^182^, zinc^183^, magnesium^184^, and/or carbonates^185,186^ with the surrounding environment (e.g., hydrogen peroxide^187^). For instance, the ability of zinc to produce hydrogen bubbles in acidic environments (such as a gastric fluid) was exploited to propel zinc-loaded microrockets in HCl solution with high speed^188^. Although chemical actuation enables high-speed locomotion of microrobots, the toxicity of the most common fuels (e.g., metal particles) constrains their usage in biological applications^10^. Additionally, side products of the propulsion reaction may result in an undesired pH change in tissues^63^. Biocatalytic propulsion of nanomotors, as a biocompatible alternative to toxic fuel sources, can produce power from urea and glucose solutions with the addition of urease and glucose oxidase enzymes to robots, respectively^189,190^.

##### Microorganism-based actuation

Biological swimmers, such as algae^191^, bacteria^192–194^, and sperms^195–197^, can be coupled with microrobots for actuation and control. These microorganisms swim by moving their flagella (i.e., a hair-like appendage evolved for the locomotion of microorganisms) in fluidic environments with low Reynolds numbers^10^. Reports of microorganism-enabled microswimmers are available in the literature. For instance, motile bacteria-based microrobots delivered drug cargo by following chemical gradients with a maximum velocity of ~40 μm/s^120^. Furthermore, microswimmers, propelled by freshwater green microalga, successfully moved in biological fluids, such as cell culture medium, plasma, and blood, with mean swimming speeds ranging from 50–100 μm/s^198^. Moreover, muscle cells enabled the movement of microrobots through natural contraction and expansion^199–201^. Cardiovascular cells were cultured on polymer microstructures and generated enough deformation to move the structure inside a fluid with an average speed of 81 μm/s^202^. However, the application of microorganisms is limited to biological environments with certain nutrients (as a power source for microorganisms), where precise navigation of microorganisms is challenging, and the travel range is limited. Moreover, biologically actuated microswimmers should be injected near targeted tissues (as the travel range is limited), complicating the administering process for critical sites (e.g., near cancer tumors)^10^.

#### Off-board actuation

##### Magnetic actuation

Magnetic fields are widely in use in biomedical applications, such as imaging (e.g., magnetic resonance imaging (MRI)^203,204^), magnetic sensors^205^, and diagnosis (e.g., magnetic levitation (MagLev)^206–209^). Magnetic-particle integrated microrobots can be moved by external magnetic gradients^210–212^. Likewise, RMFs can be employed to spin magnetized microrobots that have the structural ability to convert spinning into linear movement (e.g., helical microrobots)^149,213^. In addition, magnetic particles can be added to flexible structures in order to create fish-like deformations by exposing these structures to an oscillating magnetic field, resulting in controlled, flagella-like swimming motion^214,215^. Magnetization of microrobots is mostly achieved by embedding magnetic particles (e.g., NdFeB microparticles, SPIONs, and FePt nanoparticles) into the structural material or coating the robot with magnetic materials, such as nickel and cobalt^178^. Magnetic actuation of microrobots offers better navigation control and speed compared to other methods since it allows for control in higher degrees of freedom (DoF)^10,178^. Moreover, magnetically actuated devices can be locomoted for longer distances since the energy is supplied from an external source. Furthermore, the biocompatibility of magnetic actuation is more established compared to other external force sources, as they are regularly used in medical imaging facilities^176^. In this regard, currently in use MRI equipment can be used for both propulsion and imaging of the magnetic microrobots at the same time (no need for new, costly equipment), decreasing the overall cost of translation of microrobots into real-life applications^216^. Nonetheless, the need for expensive and bulky equipment for the creation of the required magnetic field is a hurdle in the development of magnetically actuated microrobots. In addition, Joule heating, generated during the formation of strong magnetic fields, can jeopardize cell viability which is a potential obstacle in the biomedical application of magnetic actuation^217^.

##### Acoustic actuation

Microrobots can also be propelled with external acoustic waves. An asymmetric structure yields a pressure gradient when subjected to ultrasound waves since its different parts have distinct responses to the wave. Using this method, metallic microrods with the ability to thrust, spin, and ascend with acoustic excitation inside a liquid can be generated^218,219^. Likewise, flagella-like flexible tail structures, that yield swimming motion upon excitation with acoustic waves, can facilitate the actuation of microdevices^220,221^. Furthermore, bubbles inside structures oscillate when subjected to an acoustic field and generate a force on the structure. This phenomenon can be utilized to selectively manipulate microswimmers in liquids^222–224^. Cells^225^ or cell imitating micromotors (e.g., cell membrane-coated micromotors)^226,227^ can also be potent biocompatible micromotors that are actuated by acoustic waves. As an off-board actuation method, acoustic actuation also has the ability to travel long distances. Moreover, acoustic waves can propel numerous microrobots in parallel with no threat to cells/tissues. In addition, it is possible to control microrobots deep inside the body since the waves can travel through tissues. However, individual control of microrobots is not achievable with acoustic actuation since ultrasound waves propagate spatially and affect all the particles they contact. Besides, available materials for acoustically actuated particles are limited and need further development^176^. Additionally, the production of mentioned microdevices is challenging and expensive due to the need for precise geometrical requirements.

##### Light-based actuation

Microdevices can also be powered and manipulated by light, such as photo-deformation, electrophoresis, or thermophoresis^228^. Photoactive liquid-crystal elastomers^229,230^ can be utilized to produce light-driven microwalkers^231,232^ and peristaltic microswimmers^233^. As an alternative, the plasmonic photocatalytic effect of gold and titanium oxide coatings can be used as a microrobot actuation method considering the generation of electrophoretic force under visible light^234^. Furthermore, exploiting the fact that exposure to NIR light creates a temperature gradient along the horizontal axis of an asymmetric structure, metallic needles can be thermophoretically propelled inside liquids^235^. Moreover, thermocapillary forces can be utilized to move microparticles by creating bubbles in fluids using light^236^. Alternatively, OTs can be employed for the purpose of manipulation of biological samples and cargo^237,238^. In order to make optical actuation feasible, possible adverse effects of certain wavelengths of laser radiation on living cells should be investigated. Although usage of light-induced actuation for in vivo applications is limited (as the penetration depth of light through the skin is limited), this method offers high spatial resolution and specificity. Nonetheless, with the use of transparent material, light-based actuation can be potential for in vitro and on-chip applications. The body penetration issue can be partially addressed by devising light-emitting microrobots (powered by an external source) that can operate/move inside tissues (actuating/leading a swarm of light-sensitive microrobots)^178^. Besides, further modification of material can enable the actuation of microrobots with light intensities similar to sunlight, resulting in operational microrobots with no need for external power sources^178^.

##### Electric actuation

Different parts of microstructures can be polarized by AC electric fields, inducing electroosmotic flows that can be used to propel microrobots^239,240^. In addition, DC electric fields can be used to trigger chemical reactions on the side of a microparticle in order to generate a force gradient that can locomote microdevices^241^. A combination of electroosmosis with electrophoresis can be employed to control nanorobots on graphene surfaces^242^. Additionally, electric-field-guided manipulation (jumping) of liquid metal micro fleas on solid surfaces was reported^243^. Furthermore, platinum-gold nanomotors can be electrically stimulated to capture, transport, and release cargo^244^. Moreover, Janus particles, suspended in liquid, can be manipulated with an electric field^245,246^. However, there is a paucity of reports for electrically actuated microrobots with biological applications, demonstrating a need for further investigation in this field.

#### Hybrid actuation

Hybrid actuation (integration of available methods) facilitates the fabrication of microrobots with a higher operational range and more responsiveness/sensitivity to certain environmental parameters (e.g., pH). For instance, magnetic actuation can help for traveling long distances inside the body, while an embedded pH-sensitive biological actuator can support targeting a certain pH range at the target region. Similarly, ultrasound propelling, along with magnetic guidance, can be used to transport bacteria accurately (223). Likewise, magnetic actuation can drive drug carriers to targeted sites/tissues, in which specific drug/dose can be released with ultrasound actuation^247^. Magnetic bacteria *Magnetospirillum* *magneticum* enables autonomous penetration to the hypoxic region of a tumor after being carried to the tissue with external magnetic actuation, enabling targeted cancer therapy with biohybrid microcarriers inside tumors^248^. Furthermore, pollutant degradation in water can be enhanced by utilizing external light powering and autonomous chemical propelling together^249^.

All in all, each of the stated actuation methods has pros and cons. The most proper method should be selected considering the proposed application (e.g., environmental or biomedical tasks), operational environment (e.g., acidity, immune system response, and temperature), desired speed/range, and available equipment (e.g., magnetic field generator or multi-spatial lasers). While microorganism-based actuation is limited to biological environments with a lack of precise navigation control capability^10^, chemical actuation can produce toxic byproducts (despite enabling fast propulsion)^216,250^. On the other hand, although acoustic actuation can propel multiple microrobots simultaneously deep inside tissue, limited material choice and the inability to control microrobots individually are pitfalls of this method^176,218,219^. Light-based methods can partially address individual control problems (using a high spatial range), whereas light penetration depth in tissue and potential cell damages are challenges to be solved^178^. To date, magnetic actuation has attracted more attention to be translated to clinical applications since currently in-use MRI systems can be used for propulsion and tracking of magnetic microrobots (i.e., no need for new, costly equipment in clinics)^216^. Moreover, magnetic actuation is one of the most explored and used actuation methods owing to the longer locomotion range, availability of equipment, ease of implementation, relatively high precision, and better maneuverability (higher DOF compared to acoustic and microorganism-based methods)^10,178^. However, the minute size of microrobots confines the total amount of magnetic nanoparticles that can be embedded in them, which limits their magnetizability and necessitates the use of bulky magnets, causing Joule heating and cell damage^217^. Furthermore, the use of 3D printers to embed more magnetic nanoparticles in microrobots is challenging as it is a trade-off between printability and nanoparticle concentration^149^.

Among available 3D printing methods, TPP/MPP offers the best printing resolution to print micro/nanorobots. However, despite immense attention to magnetic control techniques, the fabrication of magnetically actuated microrobots with TPP/MPP is challenging. The total amount of magnetic nanoparticles embedded in a microrobot is directly correlated with the magnitude of magnetization. Maximizing the volume fraction of the magnetic nanoparticles in the precursor suspension can amplify step-out frequencies, ultimately enhancing the achievable translational thrust, speed, and maneuverability^149^. Nonetheless, at lower laser intensities, high nanoparticle concentration physically blocks the propagation of the polymerization as fewer chains can be generated to complete the assigned laser trajectories, deteriorating the structural quality of the final microrobot as a consequence of insufficient polymer links^149^. While higher laser intensity can potentially penetrate concentrated precursor suspension, dense nanoparticles can interact with the laser light, causing local heating, bubble generation, and structural damage. This issue can be partially addressed by using more laser-sensitive 3D printing inks (which can be crosslinked with lower laser intensities) and/or iron-oxide nanoparticles which possess better colloidal stability, decreasing their tendency for aggregation at higher concentrations (enabling utilization of high-intensity light beams)^149^.

### Future perspective

#### Utilizing novel/smart materials in the fabrication step

Smart materials, also known as intelligent materials, can be described as materials that possess self-sensing, -actuating, and -healing, as well as signal generation and/or shape-changing abilities in response to external stimuli^251^. Stimuli-responsive polymers (e.g., some types of hydrogels, shape memory polymers, and piezoelectric polymers), shape memory alloys, biomaterials (e.g., chitosan, cellulose, and cells), and nanomaterial-based composites (e.g., fibers, ferrofluids, and magnetofluids) can be examples of the smart materials^64^. Manufacturability of these materials is another consideration for microrobots. 3D printing provides rapid manufacturing of various designs with a decent resolution (down to 10 nm using TPP). Recent advancements enabled the 3D printing of hydrogels, gelatin-based materials, collagen, and protein-based materials^252^, with the capability of printing different materials concurrently, facilitating the incorporation of different smart materials^57^. Layers of different smart materials can be used in microrobots in order to achieve behaviors, such as cooling, heat generation, propulsion, degradation, adaptation, selective deformation, sensing, environmental taxis, and healing^13,253^.

For example, microrobots were made using TPP 3D printing of polymer poly(*N*-isopropylacrylamide) (PNIPAM), with the ability to swell and shrink with changes in temperature, pH, and calcium ion while remaining magnetically maneuverable^254^. These capabilities of the robot were used to transport it through a channel with varying cross-sectional areas by reducing its size via increasing its temperature in regions that have smaller dimensions than the particle^254^. Besides, 3D printing of magnetic-particle-embedded temperature-responsive PNIPAM allowed leptocephali-like camouflage of microrobots in water with its translucent structure without affecting its locomotive capabilities^255^. In addition, magnetic nanoparticle-embedded microswimmers were produced by TPP using ChMA, a photosensitive polymer derived from chitosan, displaying an ability to unbind molecules that are bonded to them upon exposure to light^140^. In addition, biological particles can be embedded within polymers to enable new properties. For instance, the integration of synthetic proteins into soft materials enabled self-healing of the material in a few seconds upon local mechanical damages^256^. Moreover, the incorporation of bacteria with the surface of polyelectrolyte multilayer (PEM) microparticles enabled them to track chemical gradients^257^, which facilitates the targeting of certain cell groups, e.g., tumor cells^258^. In another study, as a novel material, magnetic particles were embedded in sugar-based SLS 3D-printed helical milliswimmers (Fig. 6F–H)^259^. The printed microrobots were magnetically maneuvered inside water/glycerol (W/G) with the ability to dissolve in biological fluids. Using SLS, a laser was focused onto layers of 25 wt% of BaFe_12_O_19_ microparticles containing sucrose powder to produce ABF structures. Afterward, BaFe_12_O_19_ particles were magnetized in a 22,000 Gauss DC magnetic field. A rotating magnetic field of 30 mT at 5 Hz stimulated a corkscrew motion of the milliswimmer in 30 wt% W/G (with similar fluidic properties to blood). Degradation of the ABF, after 20 min, was noticeable so that the corkscrew motion was stopped, demonstrating the ability of sugar-based microrobots for drug delivery and unhazardous disposal^259^.

#### Intelligent microrobots

##### Artificial intelligence (AI)

The AI was firstly developed as computational intelligence (CI), which employs sensing, learning, control, adaptation, actuation, and analysis of data with computation. Machine learning (ML), a subdivision of AI, is the science of enabling computers to autonomously learn from past experiences or example data without being explicitly programmed for that specific task^260–264^. ML can empower 3D printing by computational design optimization from a design database^265^. For instance, hierarchical ML was utilized on 38 test runs of printing silicone in order to boost printing speed up to 2.5 times without sacrificing print quality^266^. Moreover, a deep learning (DL)—a subdivision of ML—algorithm (convolutional neural networks (CNN)) along with a feedback loop was used for real-time detection and correction of defects during FDM 3D printing^267^. Integration of AI approaches into the 3D printing process of microrobots enables researchers to achieve optimal robot performance (e.g., by optimizing geometrical parameters and consequently drag forces) while optimizing printing time and needed material.

DL was implemented to enable simultaneous tracking as well as pose and depth estimation of the optical microrobots^268^. Precise depth estimation of microrobots was made possible by the Gaussian process regression (GPR) algorithm and Deep Residual Network (ResNet) architecture. The GPR could regress microplatforms from a small number of data sets to generate 2D planar position estimation in order to detect the area to be visualized. The pose estimation of a microrobot was achieved with an accuracy of 99.93%, while accuracies of depth and planar rotation angle estimations were reported to be 97.76 and 99.98%, respectively^268^. In another study, as a proof-of-concept study, a numerical experiment was conducted using a reinforcement learning algorithm (i.e., Q learning) to empower particles to learn how to adapt to difficult navigational tasks in complex fluid flows^269^. The point-like smart gravitactic ability of particles to acquire knowledge about their environment and build experience on it was numerically shown in order to extend the travel range of particles^269^.

A probabilistic learning approach (Bayesian optimization (BO) with Gaussian processes (GPs)) was used to find optimum actuation parameters (e.g., magnetic field magnitude, frequency, and orientation) for maximizing the stride range of a walking soft magnetic robot while reducing the number of required experiments^270^. After running 20 learning cycles applied on three separate robots without any prior information, the stride distance of each robot on a smooth plexiglass surface was improved by 86.6, 94.7, and 60.5% with respect to the unoptimized experiments. In order to discover the effect of prior information on the method, input parameters which were obtained by investigating 123 different parameter sets were fed to the first robot. Following 20 learning cycles on the first robot, the resulting parameters were used as the first dataset of the second robot, and the same procedure was repeated from the second to the third robot. The walking range was enhanced by 70.7, 73.9, and 113.3% compared to the setup without any optimization, for robots 1 to 3, respectively. Furthermore, the efficacy of the proposed algorithm to optimize walking distance on a rough sandpaper surface was demonstrated by expanding it from 0.93 to 1.15 mm after 20 learning cycles^270^.

Design optimization of robot morphologies and controllers was performed using a batch Bayesian optimization (BBO) which allowed parallel examination/optimization of various sets of morphology parameters together with the nested controller parameters^58^. The obtained morphology parameters are later used as source values in BO modeling of controller parameters, namely the frequencies, phase differences, and amplitudes of the robot’s vertical and horizontal motors, using a GP. The parallel working ability of the hierarchical process constrained BBO (HPC-BBO) method reduced the required time for the fabrication from 21 months (for standard BO) to 4 months (for optimized microrobot)^58^.

##### Physical intelligence (PI)

PI is described as “physically encoding sensing, actuation, control, memory, logic, computation, adaptation, learning, and decision-making into the body of an agent”, which is another method to improve the performance of microrobots in response to local changes in the environment^64^. Here, “intelligence” can be defined as the ability of microrobots to sense, interpret, control (predict, plan, decide, and regulate), act (coordinate and move), and learn (evolve and adapt) continuously and autonomously^64^. To make an analogy to living species, the CI is equivalent to neural intelligence, while the PI is similar to evolutional adaptations to environmental changes. PI is a more viable candidate to produce intelligent microrobots since the fabrication of intelligent devices (with CI) in micron scales with embedded on-board sensors and computational capabilities is challenging so far^64^.

PI can be developed in microrobots by employing passive or active smart materials, mechanisms, and structures with self-governing behaviors such as propulsion, adaptation, and degradation. For instance, a micro delivery device can dissolve in specific regions in response to certain enzymes^149^. Similarly, a microswimmer can make use of the presence of the light in order to thrust itself^271^. Furthermore, mechanical logical operators, mechanical memory, smart structuring (e.g., origami), or taxis behavior can be adapted to implant PI capabilities into microrobots. For example, the surgical treatment capability of an origami-inspired micro-operation device was demonstrated as a precise tool for teleoperated microsurgery with a reduced deviation from the desired trajectory by 68%, compared to manual operation^272^. Likewise, microfluidic logical operators can be utilized to enable autonomous regulation of fluid flow, resulting in controlled decomposition of on-board fuel supply to govern the locomotion of soft robots^273^. Moreover, a combination of functions can be encoded into microdevices in order to implement sensing, controlling, learning, and actuation into the same design. In this regard, a soft robot that can sense chemicals with engineered bacteria and convert this signal into electronic signals and mechanical gripping motion can be developed^274^. In addition, magnetic robots that can sense increasing flow rates and change their shape in order to prevent being carried away by a flow can be realized^275^. However, such multi-capabilities necessitate the implementation of computational optimization in order to prevent possible conflicts between different functions.

### Translation challenges

In order to translate microrobots from laboratory to in vivo clinical setups, both production and performance aspects should be considered and optimized^61^. These essential precautions include: (i) appropriate design based on the proposed application (application-specific design strategy), (ii) material selection (e.g., biocompatibility and biodegradability), (iii) manufacturing method selection (e.g., resolution, cost, fabrication and turn-around time, producibility of complex designs, and multi-material production ability), (iv) selection of suitable/effective control and actuation methods, (v) compatibility with medical imaging modalities and trackability, (vi) permeability in biological barriers inside the body, and (vii) ability to perform intended medical tasks while being able to retrieve or degraded malfunctioned microrobots in situ^61^.

Prevention of uncontrolled immune response (e.g., severe inflammation) of the host and nontoxic byproducts after biodegradation are challenges in the selection of the appropriate material for a device that is in close contact with organs/tissues^22,276–278^. To solve this obstacle, immunosuppressive mixtures such as anti-inflammatory factors can be integrated with microrobots. In this regard, an implantable 3D-printed PDMS setup was fabricated and coated with immunomodulatory hydrogels, reporting an ability to control acute and chronic inflammation over two weeks^279^. Another option for overcoming the challenge of triggering the immune system by biodegradable microrobots can be the selection of materials that their degradation residues are well-studied (i.e., known to be nontoxic to the human body), such as silk and PLA^280–282^. In addition, in the case of utilizing compound materials, the toxication impact of the additives needs to be studied separately^283^. One other consideration in this regard should be the investigation of the impacts of host response on changing the characteristics of the building material^22^. For example, functions, degradability, and compatibility of the utilized material may undergo alterations in contact with body fluids, local pH, and/or ionic content of the target site.

In addition, in clinical trials, the most manifest obstacle to the application of microrobots is the existence of various biological barriers, including tight junctions and flow/rheological barriers^61^. These biological barriers are present in the entry points of microrobots and pose challenging obstacles for microrobots’ motion and functionalities. Although novel entry routes to the human body have been proposed recently (e.g., human eyeball^284^), bloodstream, cerebrospinal fluid, and oral entry are among the most common routes for microrobots entry^61^. While all microrobots entering the body should cope with protein corona challenge, microrobots operating in blood face flow/rheological barriers, opsonization, intratumoral pressure, and fibrous matrix^61^. Besides, orally administered microrobots need to deal with gastric juice, microbiota, mucosal penetration, and lamina propria, where microrobots in cerebrospinal fluid confront the ependymal layer and pia mater^61^. Hence, the material, travel path, and actuation method of the microrobot should be planned according to the challenges a microrobot should overcome from entry to the target site.

One of the partially underestimated challenges associated with the translation of microrobots is the timely and costly pre-commercialization test procedures and regulations. As a rule of thumb, any material/device with biological purposes must be compatible with that specific biological environment^61^. Thus, based on the application proposed for a particular microrobot (e.g., drug delivery, microsurgery, imaging, and/or sampling) and the entry routes to the human body (e.g., digestion, injection, or rectal), a microrobot has to navigate/operate in different environments (e.g., blood, intestine, and/or tissues), complicating the establishment of a general standard for microrobot biocompatibility tests to ensure short- and long-term safety of a newly produced microrobot. Since there is no comprehensive standard for microrobot tests so far, the following methods can be used: (i) good laboratory practice (GLP) procedures which can provide the integrity and quality basis of the preclinical (i.e., without human subjects) research and development^285^; (ii) international organization for standardization (ISO)—Standard 10993: “Biological Evaluation of Medical Devices” and ISO 10993-1, “Biological evaluation of medical devices—Part 1: Evaluation and testing within a risk management process”, which can be used for traditional in vitro and in vivo biocompatibility tests (ISO 10993 testing strategies are acceptable in Europe and mostly in the USA)^286^; (iii) food and drug administration (FDA)—the 510(k) (premarket notification) process and premarket approval (PMA) regulatory, which reviews and clears robotic-assisted devices that are going to be in direct or indirect contact with the human body^61,286^. However, current standards stipulate timely procedure, which decelerates the translation of microrobots. For instance, an average of 10 months and virtually $31 million are needed for a device to go through the 510(k) process (from first filing (submission) to clearance)^62^. This can take over 54 months and $94 million for PMA, from first communication to market^62^. Therefore, in order to facilitate the translation of microrobots from bench to bedside, microrobot test standards should be established to guarantee utmost safety while decreasing the necessary time and costs.

### Concluding remarks

Despite advancements in diagnostic and therapeutic methods, current sampling, surgery, and treatment techniques are mostly invasive, engendering side effects post-treatment and reluctance in patients to undertake proper therapy. Microrobots can enable access to sites deep in the body without even a knife cut. Microrobots can be inserted into the body at a particular site, moved, manipulated/actuated, and removed/degraded remotely for imaging, sampling, surgical, and drug delivery purposes^61^. Although different actuation methods are proposed to control microrobots, the proper actuation method should be selected considering the desired application and trade-off between offered advantages and limitations of each method. While magnetic actuation is the most used method so far owing to its availability, acceptable penetration depth into the tissue, precise control, and easy operation, actuation of tiny microrobots necessitates strong magnetic fields with bulky equipment, through which adverse effects on cells is conceivable due to Joule heating^176,217^. Acoustic actuation is another candidate with tissue penetration capability. However, limited material choice and challenges associated with the fabrication of precise geometrical properties are the main pitfalls of the acoustic method^287^. Light-induced actuation is an accurate actuation method that is potent for future applications with limited penetration in tissue, restricting in deep-in-tissue applications^178^. On the other hand, on-board actuation methods (e.g., chemical reactions and microorganism-based techniques) do not need external power sources. Nonetheless, the limited travel range and possible toxicity of side products (in the chemical reaction method) are challenges to be addressed. Besides, on-board methods are limited to environments with specific substances either as fuel for microorganisms or reactants for embedded chemicals.

Although existing microfabrication methods (e.g., photolithography) could yield an acceptable resolution for microrobot production, demanding high proficiency in micromanufacturing for proper implementation, requiring manual steps, and cost-effectiveness are existing limitations. Additive manufacturing (i.e., 3D printing) is a promising approach, facilitating the design, prototyping, modification, and fabrication of desired geometries directly with minimum micromanufacturing knowledge, eradicating the need for third-party manufacturing companies for design iterations^81^. However, the fabrication of microrobots via 3D printing faces challenges such as biocompatibility, limited material choice, slow printing, and resolution confinements. Therefore, future studies can be focused on developing faster 3D printing methods without compromising the resolution.

Emerging technologies (e.g., smart materials, PI, and AI) can play a key role in the translation of current proof-of-concept microrobots to commercial clinical devices. Using smart material, PI can be realized by encoding sensing, actuation, and learning/decision-making abilities into microrobots to independently respond to local changes in their environment^64^. In addition, AI can be applied both in the design process (to optimize dimensions based on the defined application and environmental factors), and actuation process (to find optimum trajectory and actuation parameters), ultimately improving imaging/therapeutic efficacy and cost-effectiveness. Furthermore, a comprehensive test procedure should be designed specifically for microrobots to truncate current test methods while guaranteeing safety.
